# Video Modeling and Social Skills Learning in ASD-HF

**DOI:** 10.3390/children7120279

**Published:** 2020-12-08

**Authors:** Alessandro Frolli, Maria Carla Ricci, Antonia Bosco, Agnese Lombardi, Antonella Cavallaro, Francesca Felicia Operto, Angelo Rega

**Affiliations:** 1 DRC—Disability Research Centre, University of International Studies of Rome, 00147 Rome, Italy; 2 FINDS—Italian Neuroscience and Developmental Disorders Foundation, 81040 Caserta, Italy; mariacarla.ricci1@gmail.com (M.C.R.); ant.bosco@hotmail.it (A.B.); lombardiagnese@gmail.com (A.L.); a-cavallaro@live.it (A.C.); 3 Department of Child Neuropsychiatry, University of Salerno, 84080 Fisciano, Italy; opertofrancesca@gmail.com; 4 Department of Psychology, University of Naples, 80100 Naples, Italy; angelo.rega@unina.it

**Keywords:** peer video modeling, self-video modeling, ASD-HF, adolescents, social skills, mirror neurons

## Abstract

Autism spectrum disorders represent a heterogeneous group of clinical situations, and are mainly represented by a deficit of social communication. In this study, we compare two strategies to enhance communicative/social skills, namely self-video modeling and peer video modeling. The subjects were divided into two groups treated via the method of self-video modeling (group 1) or peer video modeling (group 2). For both groups of subjects affected by ASD-HF (Autism Spectrum Disorder-high-functioning), three different activities were proposed: (a) interacting with a salesperson while making a purchase, (b) initiating and maintaining a conversation with peers, and (c) starting and maintaining an enjoyable activity with a peer. The ability to rapidly accomplish the task was used as the main criteria to appraise the groups’ responses to the proposed activities. In group 1, the use of self-video modeling procedures demonstrated a faster and correct execution of the three proposed tasks (especially task 3) when compared to group 2. In group 2, the use of peer video modeling has instead led to a slower acquisition of abilities to process and perform the tasks. The use of self-video modeling speeds up the acquisition of skills to perform communicative/social tasks, compared to peer video modeling’s slower performance in subjects with ASD-HF. Results could be related to either the amount of time the subject is exposed to the task or to the capacity of ASD-HF subjects to self-value one’s own actions more than others. In our work, we have tried to reset the differences in exposure time. Therefore, self-video modeling is demonstrated to be more effective, as it produces a response to the signification/mirroring characteristic of ASD-HF.

## 1. Introduction

One of the two descriptive diagnostic criteria of autism spectrum disorder in the (Diagnostic and statistical manual of mental disorders) DSM 5 (2013) [1] is represented by the social communication deficit. A qualitative and quantitative alteration in this area includes a vast spectrum of clinical manifestations, for instance, in the most severe cases, subjects with milder impairment show a lack of language learning skills and scarce conversational skills, along with a total absence of promoting social interaction and a reduced ability to share interests, affections, and emotions. The elimination of different diagnostic subcategories and the inclusion of multiple heterogeneous clinical pictures in a single diagnostic category made it necessary to introduce dimensional qualifiers. Subjects with no cognitive difficulties and language delay, previously diagnosed as Asperger’s Syndrome, are in the category range of mild clinical severity of symptoms (level 1). In these subjects, the deficit is mainly communicative/social; therefore, it becomes a priority to work on these social skills to ensure real inclusion [2]. In fact, many children with mild autism are more prone to develop secondary forms of social anxiety and depression related to the difficulties of building relationships with peers [3]. A variety of intervention models are currently being used for the development of social skills in high-functioning (HF) ASD: behavior modification techniques, peer tutoring strategies, the use of social stories, PRT (pivotal response training) strategies, and the use of video modeling [4,5,6,7]. Video modeling exploits the fact that subjects with ASD are motivated by the attraction towards new technologies, such as tablets, smartphones, and PCs. These devices could facilitate the teaching of social skills in the form of peer video modeling (PVM) and self-video modeling (SVM) in subjects affected by ASD-HF. Video modeling is a method used by applied behavior analysis (ABA) to teach a variety of competences: independence, play activities, and communication [8]. There are numerous studies in the literature that describe the effectiveness of this strategy even for complex social tasks: acquisition of conversational skills [9,10]; acquisition of the ability to comment and compliment [11]; acquisition of pragmatic skills [12]; and acquisition and enhancement of the ability to initiate and sustain social relationships [6,13,14,15]. Usually, the VM is structured in such a way that the child can watch a recorded video of a specific task to develop the gradual ability to perform the task independently [16]. The video clearly highlights the instructions and basic stimuli to perform the task [17], the sequence of antecedent environmental stimuli, the emitted behavioral response, and the environmental consequences [6,14]. In PVM, the subject of the video is represented by a peer, and is instructed to clearly carry out the task. On the contrary, in SVM, the subject of the video is represented by the trainee. Thus, the subject can look at himself in the video while performing the task. When recording the video, the subject is prompted with continuous instructions. In the present study, we wanted to compare SVM and PVM strategies to teach children with ASD-HF three different social tasks. The chosen tasks are: (a) interacting with a salesperson while making a purchase, (b) initiating and maintaining a conversation with peers, and (c) starting and maintaining an enjoyable activity with a peer. Specifically, in this study, we wanted to compare the two types of treatment, and the performance time for acquisition of the proposed social tasks. The hypothesis tested in this work refers specifically to the ability of SVM to allow faster acquisition. In the ASD-HF scenario, these differences would be supported by a higher SVM response to the capacity of signification/mirroring when actions are self-performed versus a deficit of decentralization and mirroring compared to other performed actions [18,19,20,21,22].

## 2. Materials and Methods

### 2.1. Participants

In this study, we considered 60 subjects who had received a diagnosis of ASD level 1 (DSM 5, 2014) and divided them into two groups. All of the subjects had been recruited from the same city (Caserta) with a homogeneous familiar sociocultural background. Family/environmental background was not an influencing factor on education level in either group. All subjects undertook the Wechsler Intelligence Scale for Children (WISC IV) [23] to exclude any subjects with impaired intellectual abilities and therefore a comorbidity with intellectual disability. Therefore, the inclusion criteria were as follows: (a) age between 12 and 15 years, (b) diagnosis of level 1 autism spectrum disorder in the absence of nosographically defined comorbidities, and (c) IQ ≥ 95. After the diagnosis confirmation and the sample inclusion, we divided the subjects into two experimental groups, each one comprising 30 individuals. The subdivision was random (randomized); the subjects of both groups had the same inclusion criteria and did not have different sociocultural factors. The two groups underwent two different types of treatment, as will be discussed in the next section. The first experimental group was composed of 30 subjects (20 males and 10 females) with a mean age of 13.5 years and standard deviation (±SD 0.70); the IQ total average corresponded to 104.22 (±SD 12.02). The second experimental group consisted of 30 subjects (24 males and 6 females), with a mean age of 14 years (±SD 0.92) and an IQ total average of 104.83 (±SD 7.07). The data was collected at the FINDS Neuropsychiatry Outpatient Clinic by licensed psychologists in collaboration with the Federico II Department of Psychology and the University of International Studies of Rome (UNINT). Informed consent was obtained from all participants included in the study. All procedures performed in studies involving human participants were in accordance with the ethical standards of the institutional research committee and with the 1964 Helsinki Declaration and its later amendments or comparable ethical standards.

### 2.2. Instruments

In this study, two main procedures were used:

Peer video modeling (PVM). For each stimulus situation, three videos were recorded, with an average duration of 5 min. In the video, a peer shows the four essential behaviors of each task, emphasizing the target behaviors and socially reinforcing the correct execution.

Self-video modeling (SVM). For each stimulus situation, three videos were recorded, with an average duration of 5 min and a naturalistic setting. In the video, the subjects were filmed while performing the required tasks. These subjects were provided from time to time with verbal and written prompts to guide the correct behavior to be delivered. Such prompts were not subject to resumption.

**Figure 1:**
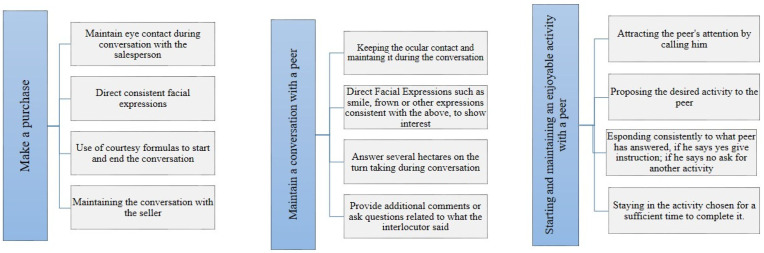
Subdivision of tasks.

In both cases, three regular social situations were identified: (a) interacting with a salesperson while making a purchase, (b) initiating and maintaining a conversation with peers, and (c) starting and maintaining an enjoyable activity with a peer. Each task was divided into four fundamental skills (Table 1); a score from 0 to 2 was then assigned to each task, where 0 indicated the absence of the behavior and 2 indicated the correct execution of the behavior, spontaneous and in the absence of assistance. Score 1 indicated that the behavior had been performed only partially correctly and/or with assistance. In particular, as regards the task of interacting with a salesperson while making a purchase, the following were considered: (a) interaction and capacity to engage (e.g. eye contact), (b) evidence of coherence using non-verbal communication, such as facial expressions, (c) capability to engage in conversation with a salesperson, and (d) use of polite behaviors and manners. Regarding the task of initiating and maintaining a conversation with peers, the following were considered: (a) interaction and capacity to engage (e.g., eye contact), (b) evidence of coherence using non-verbal communication, such as facial expressions, (c) showing spontaneous interest, and (d) engaging in conversation respecting turns. With regard to the task promoting and participating in a pleasant activity with a peer, the following were considered: (a) attracting the attention of the peer by taking initiative, (b) proposing a playful activity by taking initiative, (c) the ability to respond consistently throughout the activity, and (d) the ability to participate in the activity for at least 10 min (Table 2 and Figure 1).

### 2.3. Procedures and Tasks

Before starting the training, all of the students were assessed independently in naturalistic contexts: in particular, the skills related to completing a purchase were assessed in different settings (clothing stores, bars, restaurants, etc.). Conversational skills were assessed in a school setting while interacting with a peer during recess. The skills related to starting and maintaining a playful activity with a peer were carried out in the child’s home following an invitation from a friend. The starting condition of the subjects of the two groups was a zero score on all three tasks.

Training: Each child underwent three weekly video modeling sessions for each task lasting 5 min each. The treatment lasted a total of six weeks for the first skill, eight weeks for the second skill, and 10 weeks for the third skill. In particular, the 5-min training took place in the children’s homes, and consisted of exposure to PVM or SVM depending on the experimental group to which they belonged. For each task, during the week and on days other than the video modeling exposure, three opportunities were provided to perform the task. These opportunities each lasted 10 min. The three tasks, regarding both the administration of the video modeling and the realization of the task, were distributed between them in separate moments. In each run, the trainer measured the target behaviors on the basis of the Likert scale described above.

Therefore, the different tasks were evaluated weekly in both groups to verify the acquisition of each task, with a task acquisition condition corresponding to a score of 2 for all parts of the task. In this way, for each subject, the acquisition time of the different tasks was assessed, expressed in weeks from the start of the training.

To avoid a longer exposure time to the task in the subjects of group 1 (SVM), the subjects of group 2 (PVM) for each task had also performed a preliminary simulation of the task prompt and were recorded as in the preparation of the videos of group 1. This made the two groups identical at the start of treatment, because they both received instructions and prepared videos before being exposed to training with the SVM or PVM.

## 3. Methods

Data analysis was performed using SPSS 25.0 [24] statistical survey software. Significance was accepted at the 5% level (α < 0.05). Group averages were compared using the Student *t*-test, a statistical parameter test that can be used with two compared groups that are independent from each other. Specifically, we used the *t*-test for paired samples to make comparisons between groups, with two-tailed significance. In this study, we performed the t-test to compare the scores between group 1 (SVM) and group 2 (PVM) and evaluate the acquisition times of the three tasks: acquisition task (AT) 1 was defined as the task of buying a desired object in a shop while interacting with a salesperson, AT 2 was described as the task of initiating and engaging in a conversation with peers, and AT 3 was defined as the task of proposing and engaging in a playful activity with a peer.

## 4. Results

**Figure 2:**
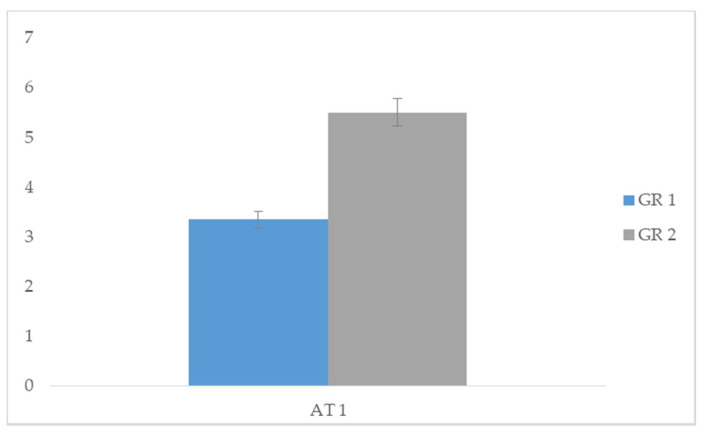
Comparison of AT1 task acquisition times between GR 1 (group 1) and GR 2 (group 2).

**Figure 3:**
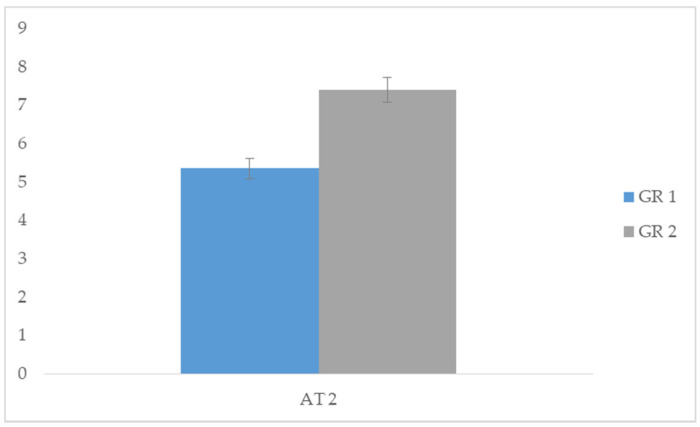
Comparison of AT2 task acquisition times between GR 1 and GR 2.

**Figure 4:**
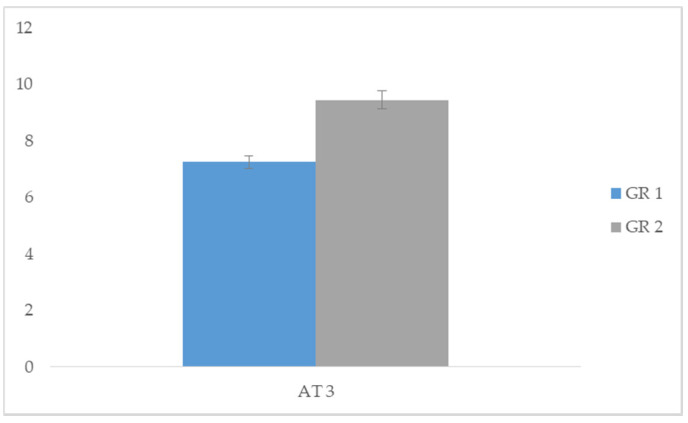
Comparison of AT3 task acquisition times between GR 1 and GR 2.

Specifically, we compared the acquisition times of AT 1 between group 1 and group 2, and significant differences emerged in group 1 (*t* = −11.831; *p* < 0.05). Data demonstrated that the task of making a purchase in a shop while interacting with a salesperson was performed in less time with the use of the SVM procedure (group 1). We then compared the acquisition times of AT 2 between group 1 and group 2, and significant differences emerged in group 1 (*t* = −11.105; *p* < 0.05). Data demonstrated that the task of initiating and maintaining a conversation with peers was acquired in less time with the use of the SVM procedure (group 1). Finally, we compared the acquisition times of AT 3 between group 1 and group 2, and significant differences emerged in group 1 (*t* = −14.139; *p* < 0.05). This data showed that the task of starting and maintaining a playful activity with a peer was acquired in less time with the use of the SVM procedure (group 1). In conclusion, the group treated with SVM showed a greater speed in acquiring the proposed tasks compared to group 2 treated with PVM (Table 3). Comparison between skills acquisition time among the different tasks are represented in Figure 2, Figure 3 and Figure 4.

## 5. Discussion

The use of video modeling in its many variants has been included among the best practices for the treatment of children with ASD and for the teaching of skills through imitative processes [25]. Several studies have been conducted in subjects with typical development to investigate differences between SVM and PVM. Various studies and some meta-analyses did not show significant differences in the effectiveness of the use of the two procedures [8,16,26,27], showing how individual differences in subjects can lead to preferring traditional PVM strategies or other SVM strategies. Other studies, on the other hand, have shown greater efficacy of PVM and SVM compared to video modeling with the adult as a model [10,15]. The number of studies that have investigated these differences in subjects with ASD-HF is limited. Most of these studies used single case designs to investigate the different degree of effectiveness of the two videos modeling strategies: SVM and PVM. In our study, we wanted to investigate on a large sample (and not on a single case) of pre-adolescents with ASD-HF which of the two procedures allowed a more rapid acquisition/stabilization of proposed social tasks. Results show significantly lower scores in the acquisition times of the proposed tasks in the subjects of group 1 (use of SVM) compared with subjects of group 2 (use of PVM). In both cases, the two procedures allowed the acquisition of the proposed tasks; the differences were related only to the speed of acquisition. Results of this study therefore confirm what emerged in the literature with respect to the effectiveness of SVM and PVM in enhancing the learning of social tasks in subjects with ASD [28], but documents a faster acquisition rate in ASD-HF subjects who use SVM. Human beings automatically imitate the actions of others and learn to understand them precisely through imitation; the imitative process allows, in fact, the construction of relationships between individuals [29]. Many social factors intervene in imitating others, such as the appearance, status, and attitudes of interaction partners, as well as our previous knowledge and beliefs [30,31]. The neural basis of imitation has been extensively studied, and is represented by the implication of the frontal cortex, the Broca area, the superior temporal sulcus, and the inferior parietal lobule. These areas are involved in a process that combines the observed movements with representations of memorized actions. The system involved in this process is called the mirror system [32,33,34]. Mirror neurons are activated when we observe or imagine a movement and when we imitate others [35,36,37]. This system is therefore involved in a bottom-up process of the sensory information to be imitated. However, the processing of the information to be imitated and the implementation of the imitation follows a top-down process. In the past, a broken mirror theory deficiency in subjects with ASD has been extensively discussed [38,39,40,41]. Currently, various studies have shown that in subjects with ASD, the mirror system is not deficient, but is the superior processing of information to be deficient; many acts are therefore imitated but not interpreted, with top-down deficits [40,42]. This processing difficulty could explain the complications children with autism have in imitating actions by giving specific meaning to them. The difficulty in processing also affects the ability to interpret the intentions of others through the tool of imitation. In fact, in the ASD-HF, the imitative processes seem automatic and controlled by sensory rather than social stimuli [43,44]; the sensory/social interference is even reduced or absent when the motor agent has a robotic form or is non-human [45]. Therefore, the salience of the social agent to be imitated (himself or a peer) in the ASD-HF could affect the accuracy of the imitative process and/or the amount of information obtained from it. In fact, in our study, the subjects that followed the SVM training showed a faster acquisition of social tasks. Studies in the literature suggest that prior knowledge of the stimuli to be imitated may influence bottom-up imitative processes [46,47,48,49]. To eliminate any differences in exposure to the task or in the amount of instructions received before the task, both groups prepared videos for an eventual SVM. Subsequently, in group 1 SVM videos were used, while in group 2 PVM videos (prepared with a peer) were used. At the time of the training, both groups had received the same amount of preliminary instruction. Therefore, reviewing the task performed by themselves in the subject with ASD-HF speeds up the acquisition of the task compared to reviewing it performed by others.

## 6. Conclusions

During the process of learning by imitation, in addition to the bottom-up component, the top-down component is fundamental. Decentralization and mirroring processes are also important in this component. The latter appear deficient in subjects with ASD-HF. Our data therefore confirm that the top-down component is fundamental in learning by imitation and that this component is deficient in ASD-HF subjects (Klapper et al., 2014). Therefore, the SVM results in speeding up the acquisition of social tasks because the subject must imitate himself rather than the other. In this way, the impact of the decentralization and mirroring deficit is reduced in the top-down component of imitation. Ultimately, the use of SVM could speed up the acquisition of social tasks precisely because it bypasses one of the main difficulties of ASD subjects: with the SVM, the deficit in the signification of the actions of others and the mirroring deficit are bypassed.

## Figures and Tables

**Table 1:** Exemplification of a task.

Maintain a Conversation with a Peer	
(1) Keep ocular contact during the conversation	0 = The subject does not maintain eye contact during the conversation 1 = The subject uses eye contact but in an inflexible way or in a limited number of exchanges 2 = The subject uses eye contact flexibly and during all conversational exchanges
(2) Direct facial expressions, such as a smile, frown, or other expressions consistent with the above, to show interest	0 = The subject does not express interest in what was said in the conversation through facial expressions 1 = The subject directs other facial expressions in a limited or exaggerated way or uses only some facial expressions (e.g., smiles in hands is contextual, but does not use expressions to express disappointment, sadness, etc.) 2 = The subject uses a wide range of facial expressions in a flexible and coherent way with the speech
(3) Answer several hectares on the turn taking during conversation	0 = The subject is unable to respect the conversational rules, for example speaking while the other is completing the speech or responding only after long pauses 1 = The subject occasionally uses non-verbal elements that allow him to understand conversational exchanges 2 = The subject can read the main forms of non-verbal communication to regulate conversational shifts
(4) Provide additional comments or ask questions related to what the interlocutor said	0 = The subject does not continue with comments or questions what the interlocutor said 1 = The subject provides additional comments on an occasional basis or not always in context with what was said by the interlocutor 2 = The subject provides additional comments or shows interest in what the interlocutor said with coherent questions.

**Table 2:** Subdivision of tasks.

**Interacting with a salesperson while making a purchase**
(1) interaction and capacity to engage (e.g., eye contact) (2) evidence of coherence using non-verbal communication, such as facial expressions (3) capability to engage in conversation with a salesperson (4) use of polite behaviors and manners
**Initiating and maintaining a conversation with peers**
(1) interaction and capacity to engage (e.g., eye contact) (2) evidence of coherence using non-verbal communication, such as facial expressions (3) showing spontaneous interest (4) engaging in conversation respecting turns
**Starting and maintaining an enjoyable activity with a peer**
(1) attracting the attention of the peer by taking initiative (2) proposing a playful activity by taking initiative (3) Ability to respond consistently throughout the activity (4) ability to participate in the activity for at least 10 min

**Table 3:** Comparison of acquisition task (AT) between group 1 and group 2.

	Group 1		Group 2			
	Means	SD	Means	SD	*t*	*p*
AT 1	3.35	0.489	5.50	0.513	−11.831	0.000 *
AT 2	5.35	0.489	7.40	0.503	−11.105	0.000 *
AT 3	7.25	0.444	9.45	0.510	−14.139	0.000 *

* p < 0.05.
